# Hospital and Health News

**Published:** 1924-12

**Authors:** 


### HOSPITAL AND HEALTH NEWS.

### New Infirmary at Chelmsford.

A new infirmary, to cost £25,000, is to be built at Chelmsford, for which a tender has been accepted.

### An Electric Operating Knife.

An operating knife that cuts human tissue bloodlessly by means of an electric current was recently demonstrated in the operating rooms of the Broad Street Hospital, New York.

### Royal Visit to Queen Mary's Hospital.

Prince Henry, president of Queen Mary's Hospital for the East End, has opened the new out-patient wing which forms the West Ham Borough War Memorial.

### Two Great Bazaars.

The bazaar on behalf of the Victoria Hospital, Blackpool, has raised a sum of £25,508. Within three days some £60,000 has been raised in Glasgow by a bazaar for the Royal Samaritan Hospital for Women towards the £70,000 desired.

### The Prince at Westminster.

On November 12 the Prince of Wales, in his capacity of president, visited Westminster Hospital to see the extensions and improvements recently made with the £80,000 collected entirely from the public.

### The Hospital Spirit.

The final ceremony has completed the war memorial gift of the Borough of St. Marylebone to the Middlesex Hospital, and seven beds have been fully endowed thereby. This is the latest instance of the hospital spirit in the district which the associated hospitals within its borders, by a common policy, have done so much to arouse, foster and extend.

### The Church Army.

Christmas, which should bring bright fires, comfort and good cheer, threatens to be one of cold and hunger to hundreds of destitute poor in London and the great towns, unless their more fortunate fellow citizens help them. This can be done through the Church Army by sending a cheque to Preb. Carlile, Hon. Chief Secretary, at the Church Army Headquarters, Bryanston Street, W. 1.

### "Winter's Pie."

Mr. R. Andrew Spottiswoode has again succeeded in obtaining contributions by many distinguished authors and artists to his entertaining annual, *Winter's Pie*, including Sir Basil Thomson, who describes what would happen "If Sherlock Holmes Came to Scotland Yard," Mr. Pett Ridge, Mr. Lawson Wood, Mr. Heath Robinson, and Mr. A. W. Bateman. *Winter's Pie* is published at 1s. 6d., and its purchase benefits not only the reader, but the charities on behalf of which it is issued.

### Closing of a Pensioners' Hospital.

Moir Park Voluntary Continuation Hospital, Preston, for naval and military pensioners has been closed. The patients are diminishing in numbers; the residue are mainly chronic; and the policy of the Ministry of Pensions is to concentrate them in larger hospitals, where they can be treated with greater economy, and, it is officially believed, with equal advantage. The hospital started in January, 1915, and was mainly composed of temporary buildings.

### Northampton General Hospital.

The Treasurer of the Northampton General Hospital, Mr Percy H. Page (Provincial Bank, Ltd., Northampton), and the Secretary-Superintendent, Mr. H. St. John Wood, are appealing for money to increase the hospital's annual income, or to enable it to build and equip a sorely-needed Nurses' Home. Under the heading of "Hospital Men of Mark" we last month described in some detail the admirable work that is being done by this important hospital.

### Tribute to the London Fever Hospital.

Dr. Weismann, Secretary of State at Berlin, while a member of the German Delegation to the London Conference, was taken ill with scarlet fever. He writes to the *Times*: "I was admitted to the London Fever Hospital, Liverpool Road, Islington, and in consequence of some serious complications had to remain there for two months. I feel deeply thankful for the kindness, the excellent medical attendance, humane care, and best possible general treatment I met with from doctors, nurses, and the whole staff of the hospital. I therefore take the liberty of addressing these lines to you in order to give public expression to my sincere gratitude to everybody connected with the London Fever Hospital."

### Improved Visitors' Rules.

The new visiting days in the Sheffield Royal Hospital, designed to replace the two former consecutive, and therefore inconvenient, week-end days, are further welcome because the production of a railway ticket now allows special entry at almost any time to suit the traveller wishing to catch a train home. In the nursery ward, naturally, restrictions remain to guard against the possibility of infection.

### Appeal by Wireless.

The Birmingham and Midland Hospital for Diseases of the Nervous System, desiring to double its present number of beds (thirty at Bath Row), has issued an appeal. The waiting list complained of by the hospital's friends is to be noted, for it was once feared by some that the demands upon it would not be great. A member of the hospital's ward has given at least one talk by wireless on the work and needs of the institution.

### Training V.A.D.s in Military Hospitals.

It is officially stated that arrangements have been made for a short course of annual training in military hospitals for a limited number of Voluntary Aid Detachment Nursing Members. Instructions as to joining will be issued in each case from the War Office. No pay will be issuable for the period of training, but those attending the course will have board and washing allowance at the rate applicable to members of Queen Alexandra's Imperial Military Nursing Service.

### The "Cripples' Journal."

The second number of the *Cripples' Journal* provides a certain amount of general as well as specialist reading. There is an article on "The Cripple on Literature." Mr. Morley Roberts contributes a little essay, which is to be followed by other literary vignettes from well-known authors, and the popular writer of girls' stories, Miss Dorothea Moore, is editing a column for children in the wards of the Shropshire Orthopædic Hospital. A rather more precise index would be a valuable addition to this excellent magazine.

### Hospital for Epilepsy and Paralysis.

The Committee of Management of the Hospital for Epilepsy and Paralysis, Maida Vale, W., have resolved to place the facilities of their Pathological Laboratory, X-ray and Swedish Remedial Exercise Departments at the disposal of medical practitioners for their patients who are not in a position to pay the usual charges. In accordance with the principles of the hospital these payments will be voluntary. Dr. McAlpine is acting as honorary pathologist until the use of the laboratory justifies a full-time paid pathologist. Communications on the subject should be sent to the secretary.

### The Bart's of New Zealand.

The Board of the Auckland District Hospital, New Zealand, has lately evoked criticism against itself by adding extensively to its existing building, thereby making the institution as large at St. Bartholomew's Hospital. There are apparently many who would have preferred the care of the sick to be less centralised. "For," it is pointed out, "though there are obvious gains such as specialised medical attention and economy of administration, there is a point at which size becomes an embarrassment and a danger. A big hospital is in peril of becoming unwieldy and disorganised."

### The "Handywomen" of Ireland.

In response to a request made by Mr. McCarron, Secretary to the Local Government Minister, the Irish Nurses' and Midwives' Union has forwarded to him lists of untrained women who are practising midwifery in the various counties in Ireland and in the City of Dublin. Mr. McCarron states that steps will be taken at once to have such persons prosecuted under the Midwives Act of 1918. As it is to the public interest that these "handywomen," who are largely responsible for the high rate of infant mortality in Ireland, should be driven out of practice, information as to their activities from anyone interested should be sent to the secretary of the Union, 29 South Anne Street, Dublin.

